# Comparison of Starts and Turns between Individual and Relay Swimming Races

**DOI:** 10.3390/ijerph18094740

**Published:** 2021-04-29

**Authors:** Xiao Qiu, Blanca De la Fuente, Alberto Lorenzo, Santiago Veiga

**Affiliations:** 1 Health and Human Performance Department, Universidad Politécnica de Madrid, 28040 Madrid, Spain; qiuxiao0646@gmail.com; 2 Sports Department, Universidad Politécnica de Madrid, 28040 Madrid, Spain; alberto.lorenzo@upm.es; 3 High Performance Center of Sierra Nevada, 18196 Granada, Spain; caynzos@gmail.com

**Keywords:** competition, kinematics, underwater swimming, video analysis

## Abstract

The present study investigated swimmers’ performances on the starting and turning segments between individual and relay races. A total number of 72 race performances of the same swimmers in both relay 4 × 100 m finals (freestyle, medley, and mixed freestyle) and individual 100 m finals or semi-finals (butterfly, breaststroke, and freestyle) from the LEN European Swimming Championships were compared with repeated measures MANOVA. Swimmers performed 5–7% faster starts in the relay than in the corresponding individual events, despite no differences in the flight phase and a lower performance (shorter distances and slower velocities) on the underwater start section. The 15 m turn times were slower in the butterfly relay races although no specific differences in the underwater parameters were observed. These results suggest that specific training of the starting and turning segments should be performed under relay conditions to optimise pacing and performance in the underwater sections.

## 1. Introduction

Recently, the world governing body of competitive swimming (Fédération Internationale de Natation, FINA) included the mixed relay events, the 4 × 100 m mixed medley, and freestyle relays in the programme of the World Championships. In addition, the International Olympic Committee accepted the 4 × 100 m mixed medley relay onto the programme of the Olympic Games. The result of these actions has been an increase in the total medal statistics of relay events at the major international swimming meets.

Relay races present specific features different from individual events, especially for relay swimmers placed second to fourth within their team. Instead of starting immediately after the referees’ acoustic signal, in a relay race the outgoing swimmers are allowed to move earlier on the block, anticipating the timing of change-over with the incoming teammate. Indeed, change-over times are related to the relay team performances [1,2], although it has been acknowledged that change-over times could hinder some other key parameters such as the force production on the block [3]. From a biomechanical point of view, individual and relay starts present some spatiotemporal differences in the swimmers’ centre of mass during the aerial phase. However, no differences in take-off velocities have been observed [4]. Additionally, the kinematic start parameters related to 5 m performance times do not coincide between both techniques as the change-over times, take-off height, and entry distance are largely related to the start performances in relays [4].

Different performance times at 15 m between the individual and relay start events could even represent differences in the overall race performance. For example, 100 m relay legs during world-level competitions were faster than the corresponding 100 m individual performances and differences were mainly explained by different block times [5,6,7]. The psychological aspects of competing on a team could also influence performances as swimmers’ motivation may be closely associated with their position within the team [8,9,10]. Indeed, relay participation could even affect the pacing strategies employed by swimmers. In a recent study, McGibbon and co-workers [11] found that half of the investigated 200 m swimmers in fourteen international competitions changed to a positive pacing strategy in relay races that impaired their swimming performance during 4 × 200 m relays.

Specific features of relay events also include differences in the underwater swimming sections. Atkison [12] found lower effectiveness in the underwater phase of relay starts unless a specific training regime oriented to the flight and entry phases was conducted. In the last few years, the role of the underwater swimming sections in overall performance has been notably highlighted from race analysis data. While being underwater after diving (starts) or pushing off the wall (turns), swimmers can maintain greater forward velocities [13,14] and reduce hydrodynamics [15,16] compared with the free swimming section. Indeed, this section has shown a meaningful impact on the 100 m and 200 m finishing times during World Championships [13,17]. To date, no previous study examined the underwater swimming sections in relay events [18]. It remains unclear whether relay swimmers would present a different behaviour during underwater sections that could impact their start and turn competitive performances.

In the present study, we aimed to compare the starting and turning segments of elite swimmer participants in the individual and relay races of the same competition (the LEN European Junior Swimming Championships). It was hypothesised that in relay races swimmers could present a superior performance in the starting segment due to shorter block times, but this would not necessarily reflect differences in specific start subsections or in the turns.

## 2. Materials and Methods

### 2.1. Participants

Swimmers who participated both in the 100 m individual and 100 m relay races of the same stroke during the 2017 European Junior Swimming Championships (Netanya, Israel) were sampled as participants. In this competition, 4 × 100 m relay final (freestyle, medley, and mixed freestyle) and 100 m individual final or semi-final footages were collected from the SPIIDEO system. Only data on the second, third, and fourth relay swimmers were recruited in the database due to the start technique of the first relay leg being the same as in the individual race. Consequently, a total number of 72 race performances (36 in individual races and 36 in relay races; 15 males and 21 females) corresponding to three strokes (butterfly, *n* = 20; breaststroke, *n* = 22; and freestyle, *n* = 30) were analysed. The mean times for the individual and relay performances in each stroke were: 53.36 ± 1.28 s and 60.10 ± 0.59 s in male and female butterfly events, respectively; 61.43 ± 1.34 s and 69.63 ± 0.54 s in male and female breaststroke events, respectively; and 49.40 ± 0.40 s and 56.00 ± 1.02 s in male and female freestyle events, respectively. Procedures were approved by the local Institutional Research Review Board in accordance with the Helsinki Declaration (WMA, October 2013).

### 2.2. Procedures

All race videos were provided by the championship organisation and sampled at 50 Hz (HD), delivering real-time multi-angle recordings. The race times (i.e., overall swim time, split time, reaction time, and change-over time) were collected from the official results website of LEN European Aquatics (http://www.microplustiming.com, accessed on 29 December 2019).

To record swimmers’ race performances in starting and turning sections, two fixed cameras (AXIS q1635, Lund, Sweden) were located at the starting (finishing) and turning sides of the pool’s public stands, respectively. Cameras were synchronised to the official timing system by the starting flash-light signal, which enabled researchers to set the time-stamp in the video analysis software (Kinovea, v. 0.8.15). The video footage of races was digitised to identify swimmers’ starting and turning movements according to the individualised distance-measured models [19]. Selected two-dimensional coordinates of the races were obtained using direct linear transformation (DLT)-based algorithms [20] to reconstruct the water surface plane. Calibration was performed through the coloured buoys of each swimming lane using eight control points uniformly distributed in the camera view. Before the commencement of each competition session, researchers confirmed the exact measurement and position of the coloured buoys in each pool lane. The video analysis procedure was completed by the same experienced analyst and very high intra-class correlation coefficients (ICCs, 0.992–0.999) were obtained.

The selected parameters of the starting segment were defined as: flight time (time duration from the swimmer’s toes take-off to the head enters the water); flight distance (horizontal distance from the starting wall to where the swimmer’s head enters the water); underwater distance (horizontal distance from where the swimmer’s head enters the water to where the head resurfaces from underwater); underwater velocity (underwater distance divided by time); emersion distance (horizontal distance from the starting wall to where the swimmer’s head resurfaces from underwater); emersion velocity (emersion distance divided by time); 15 m start time (time duration from the start signal in an individual race or the incoming swimmer touches the wall in a relay race to the swimmer’s head reaches the 15 m mark); 15 m start average velocity (15 m divided by time).

In the turning segment, monitored parameters were defined as: turn underwater distance (horizontal distance between the turning wall and where the swimmer’s head resurfaces from underwater); turn underwater velocity (turn underwater distance divided by time); turn swim distance (horizontal distance between where the swimmer’s head resurfaces from underwater and the head reaches the 15 m mark); turn swim velocity (turn swim distance divided by time); 15 m turn time (time duration from the swimmer’s feet touching the turning wall to the head reaching the 15 m mark); 15 m turn velocity (15 m turn distance divided by time). The selected parameters were previously employed in swimming race studies for individual events [21,22,23].

### 2.3. Statistical Analyses

Descriptive results are presented as mean values ± SD. Repeated measures MANOVA (multivariate analysis of variance) was conducted to examine the effect of the individual or relay performances in the starting and turning segments. The multivariate effects of the start and turn performances between different race conditions were tested using Wilks’ lambda method. Effect size (partial eta square, *η*^2^) was calculated to interpret the meaningfulness of differences and categorised as small (0.01), medium (0.06), or large (0.14) [24]. All statistical procedures were implemented using IBM SPSS Statistics 25.0 (IBM Corp, Armonk, NY, USA) and the statistical significance was set at *p* < 0.05. Data visualisation was performed using the ‘ggplot2’ package accessed via the Deducer Interface for the R software (vers. 4.0.3; R Core Team, 2020).

## 3. Results

### 3.1. Differences between Individual and Relay Events in the Starting Segment

**Figure 1:**
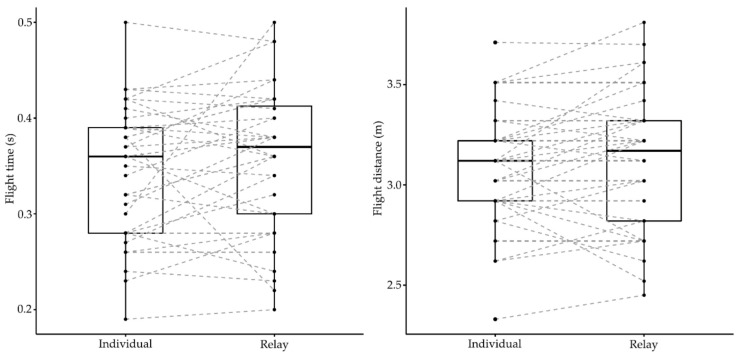
Individual and group variation in the flight start phase between individual and relay events of the LEN European Junior Swimming Championships.

**Figure 2:**
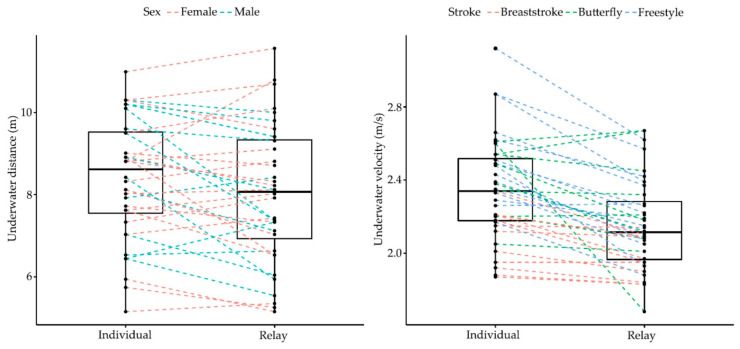
Individual and group variation in the underwater start phase between individual and relay events of the LEN European Junior Swimming Championships. Different stroke and gender groups are represented by colours.

**Figure 3:**
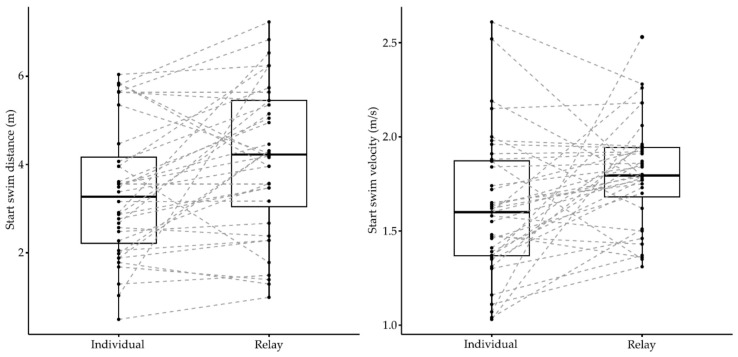
Individual and group variation in the swim start phase between individual and relay events of the LEN European Junior Swimming Championships.

Elite swimmers competing in the European Junior Swimming Championships demonstrated different start performances between individual and relay events (Wilks lambda = 0.05, F_8.00_ = 60.18, *p* < 0.001, *η*^2^ = 0.95). Differences depended on strokes (event × stroke) (Wilks lambda = 0.21, F_16.00_ = 3.36, *p* = 0.001, *η*^2^ = 0.54) and gender (event × gender) (Wilks lambda = 0.26, F_8.00_ = 8.27, *p* < 0.001, *η*^2^ = 0.74). Table 1 shows descriptive values and individual versus relay comparisons of start parameters during competitive events of the European Junior Swimming Championships. The average block times and change-over times were 0.69 ± 0.05 s and 0.29 ± 0.13 s in individual and relay events, respectively. Figure 1, Figure 2 and Figure 3 show the individual and group variation in each start phase between individual and relay events.

Differences in the start parameters were observed in all of the start subsections, except the flight time (*p* = 0.08). Compared with individual events, on average, 0.39 ± 0.01 s shorter 15 m times and 6.22 ± 0.14% faster 15 m velocities were found in relay events. There was a statistical effect of gender (event × gender) on the underwater (F_1.00_ = 7.57, *p* = 0.01, *η*^2^ = 0.20) and emersion distances (F_1.00_ = 6.22, *p* = 0.02, *η*^2^ = 0.17), indicating that male swimmers (unlike females) travelled longer distances during the starting movements in individual events. On the other hand, there was a statistical effect of stroke (event × stroke) on the underwater velocity (F_2.00_ = 3.39, *p* = 0.05, *η*^2^ = 0.18), indicating that freestyle and butterfly events (unlike breaststroke) presented faster velocities in the individual events.

### 3.2. Differences between Individual and Relay Events in the Turning Segment

**Figure 4:**
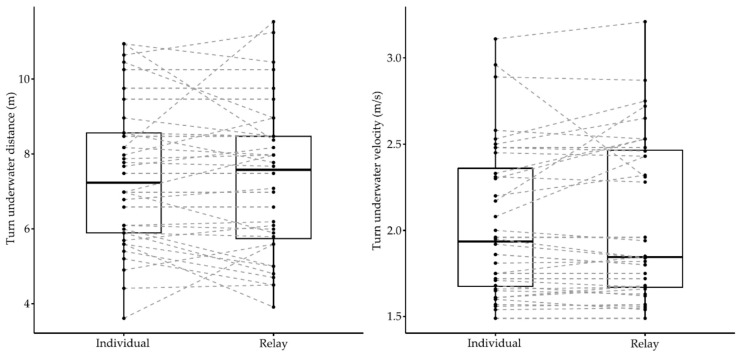
Individual and group variation in the underwater turn phase between individual and relay events of the LEN European Junior Swimming Championships.

**Figure 5:**
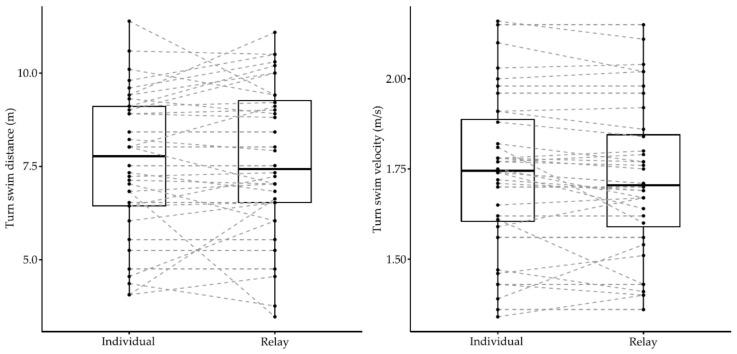
Individual and group variation in the swim turn phase between individual and relay events of the LEN European Junior Swimming Championships.

Elite swimmers competing in the European Junior Swimming Championships also showed some differences in their turn performances between individual and relay events (Wilks lambda = 0.62, F_5.00_ = 3.18, *p* = 0.02, *η*^2^ = 0.38), with differences depending on the stroke (event × stroke) (Wilks lambda = 0.52, F_10.00_ = 2.03, *p* = 0.05, *η*^2^ = 0.28). Table 2 shows descriptive values and individual versus relay comparisons of turn parameters during competitive events of the European Junior Swimming Championships. Figure 4 and Figure 5 show the individual and group variation in each turn phase between individual and relay events.

In the turn segment, differences were only observed in a limited number of parameters (15 m turn time and 15 m turn average velocity) between two events. Compared with relay events, the 15 m turn times were on average 0.05 ± 0.04 s faster and the 15 m turn velocities were on average 0.73 ± 0.04% faster in individual events. There was a statistical effect of stroke (event × stroke) on the 15 m turn time (F_2.00_ = 6.62, *p* = 0.003, *η*^2^ = 0.31) and the 15 m turn velocity (F_2.00_ = 7.04, *p* = 0.004, *η*^2^ = 0.32), indicating that butterfly events presented a faster 15 m turn time and velocity in the individual events compared with relays.

## 4. Discussion

The present study compared the starting and turning performances of elite swimmers between their individual and relay races within the same competition (the LEN European Junior Swimming Championships). Previous studies had observed similar performances of swimmers between individual and relay events apart from shorter block times in relay starts, but no information regarding the role of starting and turning segments had been reported.

In the start segment, swimmers participating in the 100 m events of the European Junior Swimming Championships globally performed 6.22 ± 0.14% faster 15 m times in relays than in individual events. This was in line with previous differences observed under experimental setup conditions [4] and it was accompanied by differences in each of the start subsections except the flight phase. The reason was potentially the different start regulations in relay versus individual events. This enabled relay swimmers to anticipate their preparatory movements on the block in order to optimise their take-off and to obtain benefits for the overall start performance [25] (pp. 50–58) [26] (pp. 59–78). Indeed, this has been reported as the main explanation for the overall performance differences observed between individual and relay efforts [6,7].

However, faster 15 m times in relay starts did not reflect a superior performance in each start section. In the flight phase, swimmers presented similar average flight distances and times when racing in individual or relay events (Figure 1), despite previous results where international-level swimmers presented longer centre of mass dive distances when performing relay starts in an experimental setup [4,12]. Although preparatory block movements on relay starts should theoretically lead to a longer flight distance [27,28], the present results suggest that swimmers do not profit from them when competing.

In the underwater phase, swimmers competing in the 100 m races of the LEN European Junior Swimming Championships apparently performed a worse underwater section in the relay starts. Specifically, male swimmers travelled 0.25 m to 1.56 m shorter underwater distances in relays and swimmers on the butterfly and freestyle strokes (both characterised by undulatory techniques while underwater) exhibited slower underwater velocities in relays (Figure 2). This is consistent with previous results showing slower start performances in relay starts if specific training was not conducted [12]. The reason for this is probably the different swimmers’ body posture and centre of mass position at the instant of take-off and water entry in relays [4], which may have several implications for the subsequent underwater swimming. Modifications of the entry width (horizontal distance from hand to feet water entry) could result in variations in the trajectory while underwater and to an increase in the drag forces [29] (pp. 10–27), which, undoubtedly, would lead to an earlier emersion [15,30]. This could be particularly important in relay starts where the control of the body position during the flight phase seems to be more difficult than in individual events in view of the great inter-subject variation observed in flight distance in the present research (Figure 1). Interestingly, female swimmers in butterfly races (unlike males) showed a similar underwater performance between their individual and relay races, probably related to their shorter flight distances and presumably better control of body position at entry (Table 1). As a consequence, their underwater distances for butterfly starts were considerably longer than for male swimmers. These results suggest that relay swimmers should adjust their entry position (entry angle) and minimise the entry width to optimally perform during underwater swimming.

In the turn segment, differences between the individual and relay races were lower than in the start segment. Swimmers competing in the 100 m events of the LEN European Junior Swimming Championships displayed faster 15 m turn times and velocities in the individual races of butterfly and freestyle strokes, respectively. However, differences at 15 m seemed to not rely on the underwater parameters but on a tendency for slower velocities when resuming surface swimming after emersion in relays (Table 2). Previous studies had observed that relay swimmers tend to swim faster in the first 50 m of the race [5,7], which could lead to a greater level of fatigue at the end of the race and, consequently, to a slower average velocity on the second 50 m lap. This could explain the observed tendency for lower 15 m turn times when racing in relays and would highlight the importance of adequately pacing 100 m relay legs. Indeed, elite swimmers do not seem to obtain a transfer of momentum when emerging from underwater to surface swimming in turns according to results in World Championships [17] unless optimal underwater swimming is being performed. Therefore, the ability to maintain the surface swimming pace after the turn in relays could be an important factor for the back-end average velocity.

This information collected and analysed regarding the starting and turning performances of elite swimmers in relay races is of special novelty as, surprisingly, it had not been previously reported in competition. However, the study sample corresponds to young swimmers (less than 18 years old) who may not completely optimise their starting and turning behaviour when competing in relay races. For example, the start and turn underwater distances obtained in the present research were shorter than those reported in previous studies with senior swimmers [13,21,31]. Therefore, caution should be taken when applying the results to more experienced swimmers. Additionally, different race contexts in the individual versus relay events may affect data. As previously observed, psychological factors related to team chances of success and race position according to rivals could influence relay swimmers’ performances [10] compared with individual races where all competitors begin at the referee’s signal. Therefore, further research taking into account the starting and turn performances of swimmers in relation to the specific race context should be addressed.

## 5. Conclusions

The present study compared the starting and turning parameters of elite swimmers participating in the individual and relay events of the same competition (the LEN European Junior Swimming Championships). Despite faster starts in relays (between 5 and 7% faster at 15 m) related to shorter block times, male swimmers in relays covered less distance underwater whereas swimmers in the butterfly and freestyle relay legs performed slower underwater swimming. In the turning segment, 15 m times tended to be slower in relays (especially in the butterfly stroke) as swimmers performed slower surface swimming after emersion. However, no specific differences in the underwater parameters were observed. These results suggest that specific training of the starting and turning segments should be performed under relay conditions to optimise pacing and performance in the underwater sections.

## Figures and Tables

**Table 1:** Comparisons of start parameters between individual and relay events in the LEN European Junior Swimming Championships.

		Male		Female	
		Individual	Relay	Individual	Relay
*Butterfly*	Flight Time (s)	0.35 ± 0.03	0.38 ± 0.02	0.34 ± 0.07	0.36 ± 0.10
	Flight Distance (m)	3.26 ± 0.20	3.34 ± 0.15	2.78 ± 0.26	2.87 ± 0.29
	Underwater Distance (m)	8.59 ± 1.23	7.03 ± 1.04 ***	9.60 ± 1.37	10.13 ± 1.37
	Underwater Velocity (m/s)	2.56 ± 0.05	2.33 ± 0.42 *	2.24 ± 0.13	2.15 ± 0.12
	Emersion Distance (m)	11.85 ± 1.14	10.36 ± 0.90 ***	12.39 ± 1.22	13.00 ± 1.34
	Emersion Velocity (m/s)	2.70 ± 0.08	3.05 ± 0.42 ***	2.33 ± 0.13	2.57 ± 0.18 *
	15 m Start Time (s)	6.09 ± 0.18	5.74 ± 0.19 **	6.86 ± 0.25	6.29 ± 0.36 ***
	15 m Start Average Velocity (m/s)	2.47 ± 0.07	2.61 ± 0.08 **	2.19 ± 0.08	2.39 ± 0.14 **
*Breaststroke*	Flight Time (s)	0.35 ± 0.07	0.40 ± 0.06 *	0.33 ± 0.07	0.34 ± 0.06
	Flight Distance (m)	3.28 ± 0.21	3.51 ± 0.24 *	2.89 ± 0.24	2.85 ± 0.18
	Underwater Distance (m)	9.82 ± 0.57	9.21 ± 0.64	8.53 ± 0.63	8.52 ± 0.44
	Underwater Velocity (m/s)	2.21 ± 0.08	2.08 ± 0.06	1.97 ± 0.12	1.88 ± 0.06
	Emersion Distance (m)	13.10 ± 0.52	12.72 ± 0.69	11.42 ± 0.59	11.37 ± 0.43
	Emersion Velocity (m/s)	2.39 ± 0.09	2.63 ± 0.08 *	2.14 ± 0.09	2.34 ± 0.08 *
	15 m Start Time (s)	6.69 ± 0.25	6.39 ± 0.25 **	7.97 ± 0.19	7.56 ± 0.17 ***
	15 m Start Average Velocity (m/s)	2.25 ± 0.08	2.35 ± 0.09 ***	1.88 ± 0.04	1.99 ± 0.04 ***
*Freestyle*	Flight Time (s)	0.39 ± 0.05	0.37 ± 0.05	0.33 ± 0.09	0.33 ± 0.10
	Flight Distance (m)	3.42 ± 0.20	3.45 ± 0.19	2.98 ± 0.18	2.95 ± 0.28
	Underwater Distance (m)	7.57 ± 1.69	7.32 ± 1.65	7.38 ± 1.41	6.88 ± 1.52
	Underwater Velocity (m/s)	2.79 ± 0.25	2.45 ± 0.14 ***	2.39 ± 0.14	2.12 ± 0.15 ***
	Emersion Distance (m)	10.98 ± 1.66	10.78 ± 1.68	10.36 ± 1.44	9.83 ± 1.45
	Emersion Velocity (m/s)	2.90 ± 0.20	3.23 ± 0.28 **	2.50 ± 0.11	2.78 ± 0.20 ***
	15 m Start Time (s)	5.91 ± 0.13	5.46 ± 0.10 ***	6.77 ± 0.13	6.46 ± 0.15 ***
	15 m Start Average Velocity (m/s)	2.54 ± 0.06	2.75 ± 0.05 ***	2.22 ± 0.04	2.32 ± 0.05 ***

Differences between individual and relay events: * *p* < 0.05; ** *p* < 0.01; *** *p* < 0.001.

**Table 2:** Comparisons of turn parameters between individual and relay events in the LEN European Junior Swimming Championships.

		Male		Female	
		Individual	Relay	Individual	Relay
*Butterfly*	Turn Underwater Distance (m)	9.28 ± 1.58	8.92 ± 1.10	8.07 ± 1.83	8.17 ± 2.10
	Turn Underwater Velocity (m/s)	1.94 ± 0.05	1.88 ± 0.07	1.68 ± 0.11	1.66 ± 0.09
	Turn Swim Distance (m)	5.72 ± 1.58	6.08 ± 1.10	6.93 ± 1.83	6.83 ± 2.10
	Turn Swim Velocity (m/s)	1.96 ± 0.14	1.94 ± 0.12	1.74 ± 0.06	1.66 ± 0.04
	15 m Turn Time (s)	7.74 ± 0.30	7.92 ± 0.27 *	8.80 ± 0.41	9.00 ± 0.31 *
	15 m Turn Velocity (m/s)	1.94 ± 0.07	1.90 ± 0.06 *	1.71 ± 0.08	1.67 ± 0.06 *
*Breaststroke*	Turn Underwater Distance (m)	9.61 ± 0.90	9.63 ± 1.19	7.69 ± 0.58	7.81 ± 0.50
	Turn Underwater Velocity (m/s)	1.75 ± 0.04	1.76 ± 0.07	1.57 ± 0.05	1.58 ± 0.07
	Turn Swim Distance (m)	5.39 ± 0.90	5.37 ± 1.19	7.31 ± 0.58	7.19 ± 0.50
	Turn Swim Velocity (m/s)	1.55 ± 0.10	1.56 ± 0.09	1.42 ± 0.05	1.42 ± 0.05
	15 m Turn Time (s)	8.97 ± 0.13	8.88 ± 0.15	10.07 ± 0.31	10.03 ± 0.36
	15 m Turn Velocity (m/s)	1.67 ± 0.02	1.69 ± 0.03	1.49 ± 0.05	1.50 ± 0.05
*Freestyle*	Turn Underwater Distance (m)	6.41 ± 0.95	6.27 ± 1.64	5.38 ± 0.93	5.18 ± 0.79
	Turn Underwater Velocity (m/s)	2.63 ± 0.40	2.70 ± 0.33	2.42 ± 0.23	2.50 ± 0.17
	Turn Swim Distance (m)	8.59 ± 0.95	8.73 ± 1.64	9.62 ± 0.93	9.82 ± 0.79
	Turn Swim Velocity (m/s)	2.04 ± 0.08	2.03 ± 0.08	1.77 ± 0.06	1.75 ± 0.05
	15 m Turn Time (s)	6.58 ± 0.15	6.68 ± 0.16	7.72 ± 0.21	7.73 ± 0.19
	15 m Turn Velocity (m/s)	2.28 ± 0.05	2.25 ± 0.05 *	1.94 ± 0.05	1.94 ± 0.05

Differences between individual and relay events: * *p* < 0.05.

## Data Availability

The data that support the findings of this study are available from the corresponding and first authors (santiago.veiga@upm.es and qiuxiao0646@gmail.com) upon reasonable request.
